# Metaproteomic Discovery and Characterization of a Novel Lipolytic Enzyme From an Indian Hot Spring

**DOI:** 10.3389/fmicb.2021.672727

**Published:** 2021-06-04

**Authors:** Dennis Sander, Yanfei Yu, Premankur Sukul, Sina Schäkermann, Julia E. Bandow, Trinetra Mukherjee, Subhra Kanti Mukhopadhyay, Lars I. Leichert

**Affiliations:** ^1^Department of Microbial Biochemistry, Institute of Biochemistry and Pathobiochemistry, Ruhr University Bochum, Bochum, Germany; ^2^Applied Microbiology, Faculty of Biology and Biotechnology, Ruhr University Bochum, Bochum, Germany; ^3^Department of Microbiology, The University of Burdwan, Burdwan, India

**Keywords:** lipase, esterase, metaproteomics, metagenomics, biocatalysis

## Abstract

Lipolytic enzymes are produced by animals, plants and microorganisms. With their chemo-, regio-, and enantio-specific characteristics, lipolytic enzymes are important biocatalysts useful in several industrial applications. They are widely used in the processing of fats and oils, detergents, food processing, paper and cosmetics production. In this work, we used a new functional metaproteomics approach to screen sediment samples of the Indian Bakreshwar hot spring for novel thermo- and solvent-stable lipolytic enzymes. We were able to identify an enzyme showing favorable characteristics. DS-007 showed high hydrolytic activity with substrates with shorter chain length (<C_8_) with the maximum activity observed against p-nitrophenyl butyrate (C_4_). For substrates with a chain length >C_10_, significantly less hydrolytic activity was observed. A preference for short chain acyl groups is characteristic for esterases, suggesting that DS-007 is an esterase. Consistent with the high temperature at its site of isolation, DS-007 showed a temperature optimum at 55°C and retained 80% activity even after prolonged exposure to temperatures as high as 60°C. The enzyme showed optimum activity at pH 9.5, with more than 50% of its optimum activity between pH 8.0 and pH 9.5. DS-007 also exhibited tolerance toward organic solvents at a concentration of 1% (v/v). One percent of methanol increased the activity of DS-007 by 40% in comparison to the optimum conditions without solvent. In the presence of 10% methanol, DMSO or isopropanol DS-007 still showed around 50% activity. This data indicates that DS-007 is a temperature- and solvent-stable thermophilic enzyme with reasonable activity even at lower temperatures as well as a catalyst that can be used at a broad range of pH values with an optimum in the alkaline range, showing the adaptation to the habitat’s temperature and alkaline pH.

## Introduction

All kingdoms of life, including animals, plants and microorganisms produce lipolytic enzymes (Carriere et al., 1994; Bhardwaj et al., 2001; Olempska-Beer et al., 2006). Their chemo-, regio- and enantio-specific characteristics make them attractive catalysts used in several industrial applications (Andualema and Gessesse, 2012). They are widely used in both, low value applications, such as the processing of fats and oils, detergents, food processing, paper and cosmetics production and high value processes, such as the synthesis of fine chemicals and pharmaceuticals (Rubin and Dennis, 1997). Overall, the global market for industrial enzymes was valued at approximately US$ 7,082 million in 2017 and is projected to reach US$ 10,519 million in 2024 (Srivastava and Srivastava, 2019). Especially solvent- and thermostable lipolytic enzymes are of industrial relevance (Gupta et al., 2004). Lipolytic enzymes can possess a broad substrate specificity, but some also exhibit chain-length selectivity and a high enantioselectivity, both traits that can be useful for certain applications (Gupta et al., 2004). Lipolytic enzymes do not require any cofactors and are often stable in organic solvents (Gupta et al., 2004). This, and the fact that they act over a wide range of pH and temperature makes them highly attractive for intensive studies (Huang, 1984; Jaeger et al., 1994; Bornscheuer, 2000; Jaeger and Eggert, 2002).

Lipolytic enzymes are classified into two major families according to their substrate specificity. The group of carboxylesterases (EC 3.1.1.3), also called “esterases,” catalyzes the hydrolysis or esterification of fatty acid esters with shorter acyl side chains (≤ C_8_) (Verger, 1997; Bornscheuer, 2002a). Conversely, the group of triacylglycerol hydrolases (EC 3.1.1.3) or “lipases” is active against water-insoluble triglycerides with longer fatty acid chains (≥ C_8_). Esterases are typically characterized by the possession of a lid structure, which moves in presence of a substrate and exposes the hydrophobic pocket and the active site (Verger, 1997; Bornscheuer, 2002a). Both groups possess a characteristic α/β hydrolase fold and contain a catalytic triad consisting of conserved Ser, His, and Asp/Glu residues (Ollis et al., 1992; Mala and Takeuchi, 2008). The Ser residue is the active site and part of a highly conserved pentapeptide motif (Mala and Takeuchi, 2008). This motif is called the catalytic elbow and consists usually of Gly–X–Ser–X–Gly, with the first glycine residue sometimes substituted by an alanine (Mala and Takeuchi, 2008).

Before the advent of the genome era, the search for novel lipolytic enzymes could be done by functional screening of microorganisms for lipolytic activity. A simple method has been described by Sierra (1957). This method uses Tween 80 in a solid medium to detect lipolytic activity. Since then, tributyrin has been introduced as substrate to screen for lipase producers on agar plates (Lawrence et al., 1967). Lipolytic activity can be detected on these agar plates by the formation of clear halos around the colonies grown (Lawrence et al., 1967). The lipases could then be purified by chromatographic separation (Lawrence et al., 1967). However, this method is time-consuming and Rappé and Giovannoni showed that typically less than 1% of the microbes of an environmental sample grow under laboratory conditions (Rappé and Giovannoni, 2003).

With the development of modern molecular biology and DNA extraction techniques, it is now feasible to screen for enzyme activity in expression libraries of the so-called metagenome (i.e., the total DNA isolated from a microbial community) (Giovannoni et al., 1990; Rondon et al., 2000). This method provides access to proteins encoded in DNA of species not growing in the lab. The isolated environmental DNA is cloned into suitable expression vectors and transformed into desired host systems. The resulting clone library can be functionally screened for activity. This method led to the discovery of numerous enzymes. Due to the typically low number of positive hits the library needs to be large, making the process time-consuming and expensive.

In an alternative and more recent approach, the DNA from a microbial community can now be sequenced using high throughput “Next Generation Sequencing” and the resulting database can easily be screened for structural motifs of known enzymes by bioinformatical analysis (Kim et al., 2004; Roumpeka et al., 2017). After the *in-silico* screening, only a reduced number of clones need to be functionally screened for activity, resulting in reduced cost and increased efficiency (Kusnezowa and Leichert, 2017; Roumpeka et al., 2017). Kusnezowa and Leichert (2017) showed the possibility to reduce the number of 6.1 million predicted proteins to only 22,566 protein families, for which they then selected a representative protein, which showed the closest homology to all other proteins in this family. Due to its targeted nature, this approach can be substantially faster than the direct library-based screening mentioned above, but also has some disadvantages: DNA *in silico* screening can only discover enzymes based on already known motifs, so truly novel enzymes or those with deviating structural properties might escape the search. Furthermore, this approach potentially detects all enzymes encoded in the DNA, not necessarily those expressed in the environment, which can be a shortcoming, too, if the habitat was selected in order to find enzymes evolved specifically to cope with the substrates present in that environment.

To address these shortcomings, we have now developed a functional metaproteomics approach in our lab, complementing the above-mentioned DNA-only approach with the immediacy of an activity-based screening on the protein level (Sukul et al., 2017). This approach has the potential to discover all expressed enzymes with desired traits in an environmental sample. For this, proteins and not only DNA, are isolated from an environmental sample. The proteins are then separated by two-dimensional (2D) polyacrylamide gel electrophoresis and, after refolding, an in-gel activity assay, based on the fluorogenic substrate *para*-methylumbelliferyl butyrate (*p*MUB) is performed. Lipolytic enzymes present in the sample hydrolyze the substrate and release fluorescent p-methylumbelliferone, which can be detected under UV light. Fluorescing protein spots are then excised from the gel, digested tryptically, and analyzed by mass-spectrometry. A DNA database, which can be created from the metagenomic sequences isolated from the very same sample is used to identify the active protein spots based on the protein mass spectra. In our initial study we were able to identify 14 lipolytic enzymes from a single environmental sample (Sukul et al., 2017). This methodology also has the potential to find enzymes with certain characteristics by screening environmental samples from habitats with selective pressure for the desired traits.

In this work, we wanted to find heat- and solvent-stable lipolytic enzymes, as high temperatures and organic solvents are often used in industrial applications of biocatalysts. Thus, we used our functional metaproteomics approach to screen sediment samples from the Bakreshwar hot spring (located in West Bengal, India). Heat-stability often equals solvent-stability (Kumar et al., 2016), and we were able to identify a novel heat- and solvent-stable enzyme in the sediment from this spring, which has a temperature of 60°C. This enzyme, termed DS-007, shows a temperature optimum at 55°C with a >40% activity range from 35 to 65°C, and high stability in organic solvents, with low concentrations of methanol increasing the activity by 40%. The enzyme is temperature stable (it retains 25% activity after 18 h of incubation at 75°C) and it has >40% activity in a broad pH range (pH 8.0–10.0). Our paper demonstrates the ability of our approach to find solvent- and heat-stable enzymes using hot springs as the source of the biocatalysts.

## Materials and Methods

### Chemicals

All chemicals were purchased from Sigma-Aldrich (St. Louis, United States). For activity assays, a Jasco V-650 spectrophotometer, equipped with a PSC-718 temperature-controlled sample holder (Jasco Corporation, Tokyo, Japan) was used.

### Sample Harvesting and Enrichment Culture

Sample collection and usage have been approved in accordance with the Nagoya Protocol on Access and Benefit Sharing by the Government of India National Biodiversity Authority under file no. NBA/Tech Appl/9/Form B/37/17/17-18/1117.

Water and mud samples were collected from the bottom of the Indian Bakreshwar hot spring and transferred into sterile vacuum insulated thermos flasks. The thermos flasks were stored in large temperature insulated Styrofoam containers and transferred back to the laboratory within 2.5 h. Subsequently, the samples were centrifuged down, aliquoted and flash frozen using liquid nitrogen. Samples were stored at −80°C for future use.

Sediment samples from the Indian Bakreshwar hot spring, with a water temperature of 60°C and a pH of 9.4, were cultivated at 37°C as an enrichment culture in M9 minimal medium with olive oil as the only carbon source similar to the methodology described in Sukul et al. (2017). After 2 days of incubation, the cells were centrifuged at 4°C and 8,000 rpm for 45 min. The pooled cell pellet was lysed by bead-beating with glass beads (0.25 mm) in *n*-Dodecyl β-D-maltoside (20 mM) and 50 mM sodium phosphate buffer (pH 7) in a ratio 10:35 (Islam et al., 2017). The bead-beating was performed for 10 min at maximum speed with a Vortex-Genie^®^ (Scientific Industries, United States). Subsequently, a methanol-chloroform precipitation (Wessel and Flügge, 1984) was performed to remove interfering agents such as nucleic acids from the protein sample. For this, 100 μL of the sample was mixed with 400 μL methanol and 100 μL chloroform. Subsequently, 300 μL water was added and the sample was centrifuged for 2 min at 14,000 g. The protein was now visible as a thin wafer between the top aqueous layer and the organic solvent layer. In the next step, the aqueous layer was removed and 400 μL methanol added. After spinning for 3 min at 14,000 g the methanol was removed, and the protein pellet was recovered in 100 μL 50 mM sodium phosphate buffer (pH 7). The sample was used directly or stored at −20°C.

### Identification of DS-007

For the 2D gel electrophoresis, 600 μg of the proteins was resuspended in 500 μL rehydration buffer. Subsequently, a commercially available IPG strip gel (GE Healthcare Immobiline DryStrip pH 4–7) was incubated with 450 μL of the proteins overnight. The isoelectric focusing (IEF) was run with the parameters described in Table 1.

**TABLE 1:** Power conditions for IEF.

Step	Volt	mA	Watt	kVh	h
1	0–500	2	300	1	2
2	500	2	300	0.01	0.02
3	500–3,500	2	300	3	2
4	3,500	2	300	57	16.3
5	60	2	300	0.1	∞

Afterward, the proteins were separated by protein size in 12.5% polyacrylamide gels. The gels were run at 10 W for approximately 5 h. Subsequently, an in-gel activity assay with fluorogenic *para*-methylumbelliferyl butyrate (50 mM) was performed to determine lipolytic activity (Sukul et al., 2017). In parallel, RuBPs staining was performed to evaluate separation quality as described (Rabilloud et al., 2001). Thirteen lipolytically active spots were detected in the protein sample. After excising the fluorescing protein spots from the gel, they were tryptically digested and analyzed by mass spectrometry. Identification was done by searching against the NCBI nr database (Release Number: 227). One protein spot was identified, termed DS-007. It showed a 100% identity with an as of yet uncharacterized putative alpha/beta hydrolase from *Ralstonia pickettii* (WP_012761013.1).

### Heterologous Expression and Purification of DS-007

A codon-optimized gene for DS-007 was designed and then ordered from GenScript (Leiden, Netherlands) in the expression vector pET-28a (++) with the restriction sites NdeI/EcoRI. This plasmid contains an N-terminal hexahistidine tag, which was fused to DS-007 and a thrombin site to cleave off the hexahistidine tag, if necessary. The plasmid was transformed into competent *E. coli* BL21 (DE3) cells via heat shock resulting in the expression strain *E. coli* DS-007. Successful transformants were selected on solid lysogeny broth (LB) medium containing kanamycin (30 μg mL^–1^). Single colonies were inoculated in LB medium (3 mL) supplemented with the appropriate antibiotics and grown at 37°C and 125 rpm for 16–18 h. Subsequently, 200 mL LB medium was inoculated with 2 mL of the preculture and incubated at 37°C until an OD_600_ of 0.6 was reached. Gene expression was induced on ice by addition of isopropyl-β-D-thiogalactopyranoside (IPTG) to a final concentration of 0.5 mM. Subsequently, the cultures were incubated at 18°C at 125 rpm for 16–18 h. The induced cells were centrifuged at 4°C and 8,000 rpm for 40 min. Supernatant was discarded and cell pellets were stored at −20°C.

For purification, cell pellets were resuspended in 5 mL 50 mM sodium phosphate buffer (pH 7.0) and sonicated on ice for three 45 s intervals with 1 min cool down periods in between (VialTweeter, Hielscher Ultraschalltechnik, Germany). After centrifugation at 4°C and 8,000 rpm for 40 min, the protein was purified using a Ni-NTA resin affinity chromatography according to the manufacturers protocol (HisPur^TM^ Ni-NTA resin, Thermo Scientific, United States). The wash and elution fractions were checked on a 4–12% Bis-Tris Protein Gel (NuPAGE^TM^, Thermo Scientific, United States) by gel electrophoresis followed by Coomassie Brilliant Blue staining. To exchange the elution buffer to 50 mM sodium phosphate buffer (pH 9.5), the protein was loaded onto an ultrafiltration vial (Vivaspin^®^ 20 Ultrafiltration Unit 10,000 MWCO PES, Sartorius, Germany) and washed three times for 30 min at 4,500 rpm with the aforementioned buffer. The total protein concentration was determined spectrophotometrically (Spectrophotometer V-650, Jasco, Japan) at A_280_ with a predicted A_280_ = 28990 M^–1^ cm^–1^ for DS-007 with hexahistidine tag (ProtParam tool, ExPASy, Gasteiger et al., 2005). The protein was aliquoted in concentrations of 12.1 mg mL^–1^.

## Gel-Based Activity Assays

To check for protein lipolytic activity, an in-gel activity assay was performed with 4-methylumbelliferone butyrate. For this, 1 μg of DS-007 was loaded on 4–12% Bis-Tris Protein Gel (NuPAGE^TM^, Thermo Scientific, United States) and the gel was run in MES-buffer at 160 V for 60 min. The gel was washed 3 times for 10 min in 2.5% Triton^TM^ X-100 (Sigma-Aldrich, United States) and 1 min in 50 mM sodium phosphate buffer pH 7.0 for protein refolding. Afterward the gel was incubated in 50 mL of the aforementioned buffer with 4-methylumbelliferone butyrate as substrate at a concentration of 50 mM for 3 min. The gel was washed once in 50 mM sodium phosphate buffer pH 7.0 and the enzyme’s activity was checked under UV-light. For quantification of the fluorescent bands the “Pixel Counter”-plugin of ImageJ2 was used (Rueden et al., 2017).

### Activity Assay

The lipolytic activity of DS-007 was determined spectrophotometrically in detail using different *p*-nitrophenyl (*p*NP) esters as substrates. A time-course measurement over 200 s was used with a data interval of 0.5 s. The enzymatic hydrolysis of substrates was followed by monitoring the release of *p*-nitrophenol at 405 nm by using the spectrophotometer V-650 (Jasco, Japan). Initial rate measurements in μM min^–1^ (*V*_obs_^Enz^) were carried out in triplicates and the data averaged by standard-deviation. The concentration of *p*-nitrophenol was determined at pH 9.5 according to Lambert Beer’s Law (ε_M pH9.5_ = 18100 M^–1^ cm^–1^). The assays were performed in a total volume of 2,000 μL using 50 mM sodium phosphate buffer (pH 9.5), 0.04% (v/v) Triton X-100 and 250 nM DS-007, unless otherwise described. The temperature was 55°C and the hydrolysis reaction was started by the addition of 50 μM of substrate. All assays were at least performed in biological triplicates and the values were corrected for autohydrolysis by monitoring the hydrolysis of *p*-nitrophenol without adding enzyme (*V*_obs_^auto^). Subsequently, equation **1** was used to correct the final initial rates (V_obs_).

(1) $${\text{V}}_{\text{obs}}={\text{V}}_{{\text{obs}}^{\text{Enz}}}-{\text{V}}_{{\text{obs}}^{\text{Au}\mathrm{to}}}$$

### Chain-Length Specificity

Chain-length specificity was measured with *p*NP-esters containing variable acyl chain lengths using *p*-nitrophyl acetate (C_2_), *p*-nitrophyl butyrate (C_4_), *p*-nitrophyl caprylate (C_8_), *p*-nitrophyl decanoate (C_10_), *p*-nitrophyl laurate (C_12_), *p*-nitrophyl myristate (C_14_), and *p*-nitrophyl palmitate (C_16_).

### Kinetic Parameters

Michaelis-Menten kinetics were determined using the preferred substrate *p*-nitrophenyl butyrate (C_4_) at concentrations between 2 and 600 μM. The measurement was carried out at optimum temperature (55°C) and optimum pH (9.5). K_m_ and V_max_ were determined using Graphpad Prism’s (GraphPad Software Inc., United States) “Enzyme kinetics—Michaelis Menten” function. k_cat_ and the catalytic efficiency (k_cat_/K_m_) were calculated based on these results.

### Determining Optimal Temperature, Temperature Stability, and Optimal pH

The temperature optimum of DS-007 was determined by varying the temperature between 20 and 70°C in 5 K steps. *p*-nitrophenyl butyrate (C_4_) was used as substrate. Short time temperature stability was investigated by incubating the enzyme for 1 h at varying temperatures between 20 and 70°C in 5 K steps. Subsequently the activity was tested with *p*-nitrophenyl butyrate (C_4_) as substrate at optimal conditions. Long time temperature stability was tested by incubating DS-007 at a concentration of 180 μM for 18 h at room temperature (control), 55, 75, and 95°C. Subsequently, 2.5 μg were loaded on a 4–12% Bis-Tris Protein Gel (NuPAGE^TM^, Thermo Scientific, United States) and the gel was run in MES-buffer at 160 V for 60 min. The gel was washed 3 times for 10 min in 2.5% Triton^TM^ X-100 (Sigma-Aldrich, United States) and for protein-refolding 1 min in 50 mM sodium phosphate buffer pH 7.0. Afterward, an activity assay was done as described in section “Gel-Based Activity Assay.” The optimal pH was determined between pH 6.0 and pH 10.0 in 0.5 pH unit steps using *p*-nitrophenyl butyrate (C_4_) as substrate. The extinction coefficients of *p*-nitrophenol at varying pH were determined for pH 6.0–10.0. A time-course measurement as described in “Activity Assay” was carried out in biological triplicates to determine the initial rates. Because of autohydrolysis of the *p*-nitrophenyl substrates the values were corrected as described in section “Activity Assay.”

### Stability in Organic Solvents and Detergents

The stability of DS-007 in organic solvents was determined using 1 and 10% (v/v) end concentration of methanol, dimethyl sulfoxide (DMSO), dimethylformamide (DMF), iso-propanol and acetone. The effect of detergents on DS-007 was determined using CHAPS and SDS at a concentration of 1% (w/v) and Tween 20, Tween 80 and Triton X-100 at a concentration of 1% (v/v). All assays were performed with 10 nM DS-007 at 55°C, in 50 mM sodium phosphate buffer pH 9.5 and *p*-nitrophenyl butyrate (C_4_) as substrate.

### Effect of Metal Ions and Inhibitors

The effect of metal ions was determined using NaCl, CaCl_2_, CuCl_2_, FeCl_2_, KCl, LiCl, MgCl_2_, MnCl_2_, and NiCl_2_ at final concentrations of 1 mM. The influence of inhibitors was determined using ethylenediaminetetraacetic acid (EDTA), dithiothreitol (DTT), ß-mercaptoethanol and phenylmethylsulfonyl fluoride (PMSF) at a final concentration of 1 mM each. All assays were performed with 10 nM DS-007 at 55°C in 50 mM sodium phosphate buffer pH 9.5 and *p*-nitrophenyl butyrate (C_4_) as substrate. 50 mM sodium phosphate buffer pH 9.5 with 10 nM DS-007 was used as a control. Reaction temperature was 55°C. To exclude artifacts due to the hexahistidine tag of our recombinantly purified protein when determining DS-007’s activity with NiCl_2_, the measurement was additionally performed with the enzyme after cleavage of the hexahistidine tag. The cleavage was done with Thrombin CleanCleave^TM^ Kit (Sigma-Aldrich, United States) according to the manufacturers protocol.

### Halotolerance

The halotolerance of DS-007 was measured using NaCl concentrations of 0.1, 0.2, 0.5, and 1 M in 50 mM sodium phosphate buffer pH 9.5. The measurement was done using *p*-nitrophenyl butyrate (C_4_) as substrate at room temperature after incubating 10 nM of the enzyme in the respective salt buffer for 3 h.

## Results

### Discovery of DS-007, a Novel Lipolytic Enzyme From Bakreshwar Hot Spring

**FIGURE 1:**
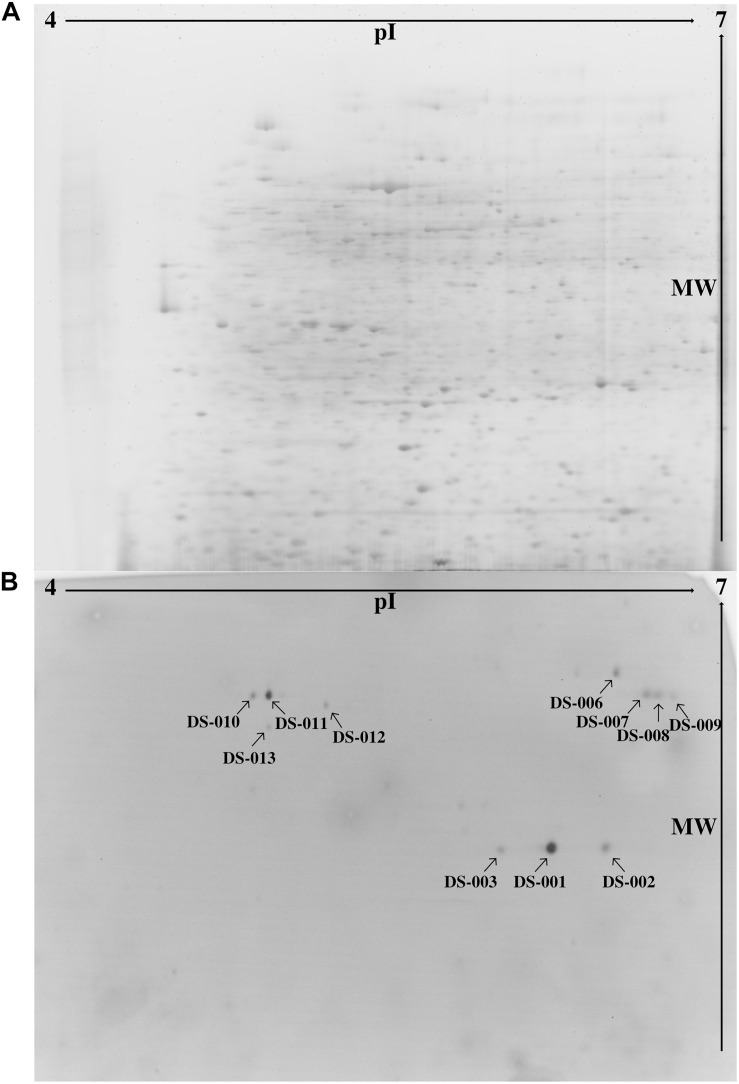
**(A)** Protein extract from an enrichment culture of sediment from Bakreshwar hot spring (located in West Bengal, India) was separated in a 2D gel and stained with RuBPS Protein Gel Stain. **(B)** 2D PAGE zymography was carried out to detect protein spots showing lipolytic activity. Present lipolytic enzymes in the gel hydrolyzed the fluorogenic substrate pMUB. The blue fluorescent *p*-methylumbelliferone was detected under ultraviolet light. Spot DS-007 could be identified by mass-spectrometry (spots marked with an arrow).

To find novel thermo- and solvent-stable lipolytic enzymes, one approach is to search habitats which would favor the evolutionary selection for the desired traits. Thus, we examined the microbiome of the sediment of the Bakreshwar hot spring, which has a temperature of 60°C and a pH of 9.4. We used a functional metaproteomics approach (Sukul et al., 2017), combining the comprehensiveness of proteomics with the specificity of an activity-based screening to evaluate the proteins produced by the microbiome of the Indian Bakreshwar hot spring for novel lipolytic enzymes. To do this, we enriched sediment samples in M9 minimal medium with olive oil as the only carbon source. After 2 days at 37°C the cultures turned turbid with a pinkish hue, suggesting bacterial growth. The bacteria were harvested, and the cell pellet lysed to get a protein extract. We then separated this protein extract by 2-dimensional polyacrylamide gel electrophoresis. Staining with RuBPS, a highly sensitive protein gel stain, revealed more than 1,500 well-separated individual spots in a pI range of 4–7 and a mass range of approximately 18–200 kDa (Figure 1A). We then performed an activity stain using the fluorogenic lipase substrate *para*-methylumbelliferone butyrate (*p*MUB). Esterases and lipases present in the gel would be able to hydrolyze this ester and the cleaved off fluorophore *p*-methylumbelliferone is visible under ultraviolet light. This means, that the resulting blue fluorescent spots indicate lipolytic activity in-gel. In total we could identify 13 lipolytically active spots under ultraviolet light (Figure 1B). After excising the fluorescing protein spots from the gel, they were tryptically digested and analyzed by mass spectrometry. Identification was done by searching against the NCBI nr database (Release Number: 227). Only one spot exhibited identity with known proteins from the NCBI nr database.

### Sequence Analysis of DS-007 Suggests That It Is an Esterase

**FIGURE 2:**
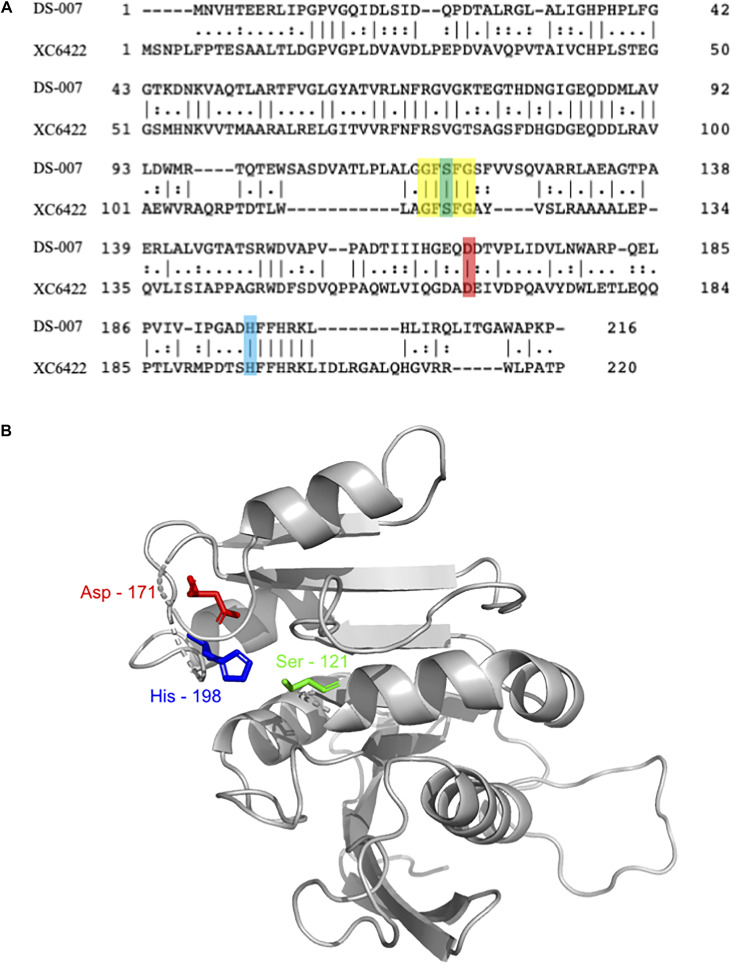
**(A)** Sequence alignment of DS-007 with XC6422 from *Xanthomonas campestris* (structure template based on this enzyme) showed an identity of 33.6% (Madeira et al., 2019). Residues forming the catalytic triad [serine, (121), aspartic acid (171), and histidine (198)] are highlighted in green, red and blue, respectively. The pentapeptide G-F-S-F-G is highlighted in yellow. **(B)** Three-dimensional structure of DS-007 was modeled with Phyre^2^ server (Kelley et al., 2015) against the d2fuka template (based on XC6422). Ser-121, Asp-171, and His-198, predicted to form the catalytic triad of DS-007 are in close proximity. The absence of a lid indicates that DS-007 is probably an esterase.

The protein contained in this spot, DS-007, showed a 100% identity with an as of yet uncharacterized putative alpha/beta hydrolase from *Ralstonia pickettii* (WP_012761013.1). This protein consists of 213 amino acids with a predicted molecular weight of 24.09 kDa. A three-dimensional structure of DS-007 was modeled using the Phyre^2^ server (Kelley et al., 2015), based on the template d2fuka. d2fuka represents the crystal structure of the protein XC6422, a member of α/β serine hydrolase without lid from *Xanthomonas campestris* (Figure 2A). The predicted structure of DS-007 revealed a typical alpha/beta hydrolase without lid (Figure 2B). The active site pentapeptide motif GFSFG is located in a so-called nucleophilic elbow, which can be seen in the structure model (Derewenda and Derewenda, 1991; Ollis et al., 1992). Like most lipolytic enzymes, the sequence of DS-007 contains a hallmark pentapeptide GxSxG with the active site Ser-121 situated in the center (residues 119–123) (Derewenda and Derewenda, 1991). Based on the Phyre^2^ Structural model, this Ser-121 was predicted to form the catalytic triad of DS-007 with Asp-171 and His-198 (Ollis et al., 1992; Bornscheuer, 2002b). To study the enzyme and its characteristics more closely, we synthesized a gene and expressed DS-007 in *E. coli*.

### Heterologous Expression and Purification of DS-007

**FIGURE 3:**
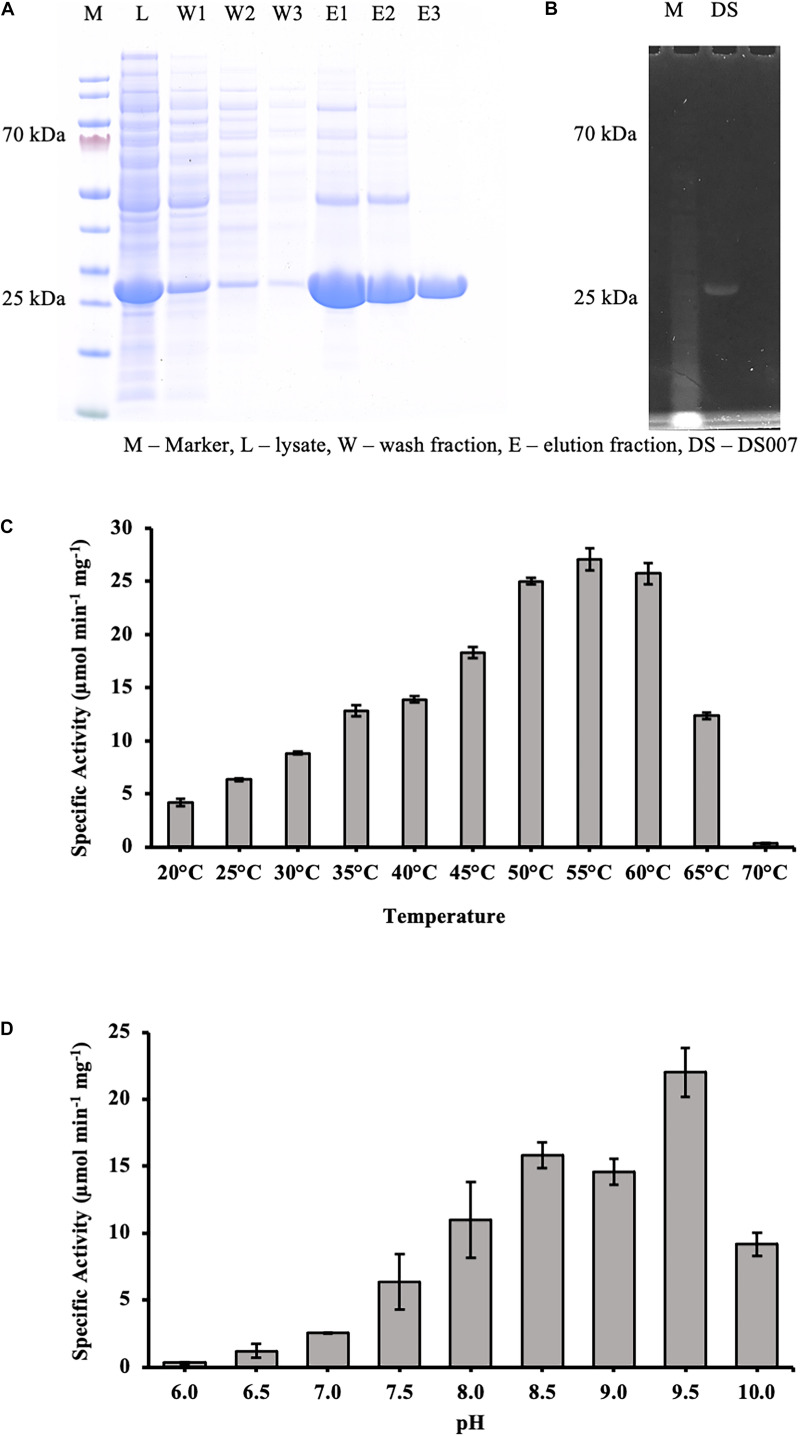
**(A)** SDS-PAGE analysis showing the purification of DS-007 by Ni-NTA chromatography. The expected size of DS-007 is 24 kDa. **(B)** An in-gel activity assay with 4-methylumbelliferyl butyrate was carried out and the fluorescent band at the position of DS-007 verified its lipolytic activity. **(C)** The temperature optimum of DS-007 is 55°C. **(D)** pH optimum was observed to be at 9.5. However, DS-007 showed good activity between pH 8 and pH 9.5. pH values lower than 7.5 resulted in a drastic loss of activity. **(A,B)** Show representative results, **(C,D)** the average and standard deviation from 3 independent experiments.

The codon-optimized gene was synthesized by GenScript (Leiden, Netherlands) and cloned via NdeI/EcoRI into the expression vector pET-28a (+), adding a N-terminal hexahistidine tag and a thrombin site to DS-007. To investigate its lipolytic activity, the hexahistidine tagged DS-007 was purified by Ni-NTA resin affinity chromatography. The washing and elution fractions were collected and checked on a 12% sodium dodecyl sulfate polyacrylamide gel electrophoresis (SDS-PAGE) followed by Comassie Brilliant Blue staining (Figure 3A). The purified protein was loaded on an ultrafiltration vial (Vivaspin^®^ 20 Ultrafiltration Unit 10,000 MWCO PES, Sartorius, Germany) to exchange the elution buffer to 50 mM sodium phosphate buffer pH 9.5. Subsequently, DS-007 was run on an SDS-PAGE and an in-gel activity assay with 4-methylumbelliferyl butyrate was carried out. A fluorescent band at the position of DS-007 verified its lipolytic activity (Figure 3B).

### DS-007 Is a Thermophilic and Alkaliphilic Enzyme

To determine the optimum temperature, we assessed the hydrolyzing activity of DS-007 against *p*-nitrophenyl butyrate at temperatures ranging from 20 to 70°C. This C_4_ substrate is similar to the in-gel substrate *p*MUB we used to discover DS-007, but instead of a fluorophore it releases *p*-nitrophenol, whose concentration can be determined spectrophotometrically. DS-007 showed the highest activity at 55°C, but still retained 40% activity at 65°C, which suggests a thermophilic profile consistent with its site of discovery (Figure 3C). We then tested DS-007’s activity at different pH values. DS-007 shows high activity in a broad range between pH 8.0 and pH 10.0 with an optimum at pH 9.5 (Figure 3D). These values were corrected with respect to autohydrolysis of *p*-nitrophenyl butyrate, which becomes significant at temperatures over 60°C and pH ≥ 10. This data indicates that DS-007 is a temperature stable thermophilic enzyme with reasonable activity even at lower temperatures, as well as a catalyst that can be used at a broad range of pH with an optimum in the alkaliphilic range, showing DS-007’s adaptation to the habitat’s pH and temperature conditions.

### DS-007 Is an Esterase

**FIGURE 4:**
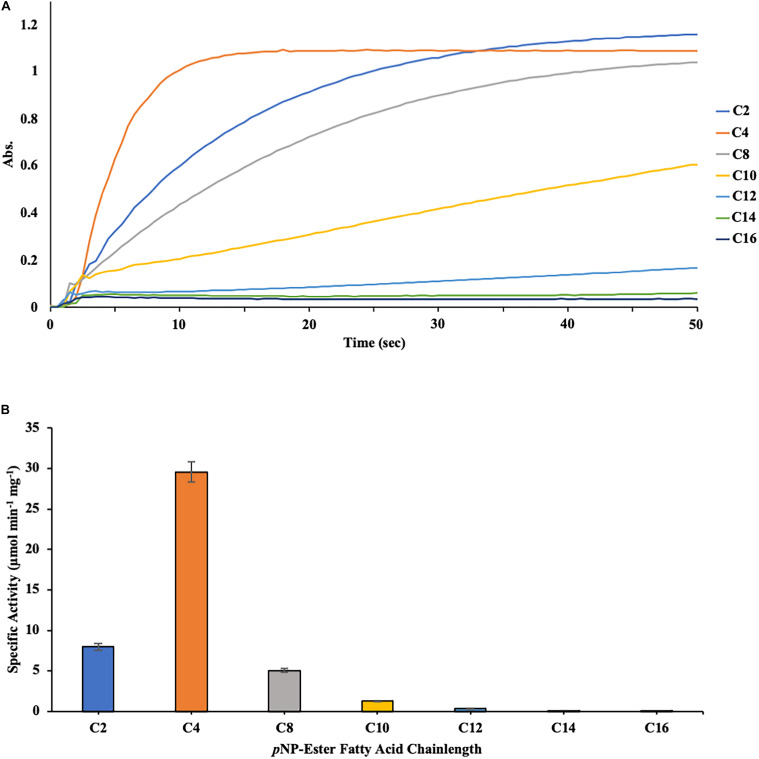
**(A)** Measurement of the initial velocity of the hydrolysis of p-nitrophenyl substrates with different chain-lengths catalyzed by DS-007. Absorption was measured for 200 s at 405 nm. This data was then used to calculate DS-007’s activity. **(B)** Substrate specificity of DS-007 showed a preference for short-chain substrates (≤ C_8_) with the maximum activity observed with *p*-nitrophenyl butyrate (C_4_). Specific activity was determined from the initial velocity determined in **(A)**. **(A)** Show representative results, **(B)** the average and standard deviation from 3 independent experiments.

The substrate specificity of DS-007 was then determined at its optimal conditions, at 55°C and pH 9.5 using *p*-nitrophenyl esters with varying chain lengths (C_2_–C_16_) (Figure 4A). DS-007 showed the highest hydrolysis activity with substrates with shorter chain length substrates (≤ C_8_) with the maximum activity observed with *p*-nitrophenyl butyrate (C_4_). For substrates with a chain length ≥ C_10_ significantly less hydrolysis activity was observed (Figure 4B). Preference for short chain acyl groups and no or little activity with long chain substrates is characteristic for esterases, consistent with DS-007’s predicted structure, which did not contain a lid (Bornscheuer, 2002a).

### Kinetic Parameters of DS-007

**FIGURE 5:**
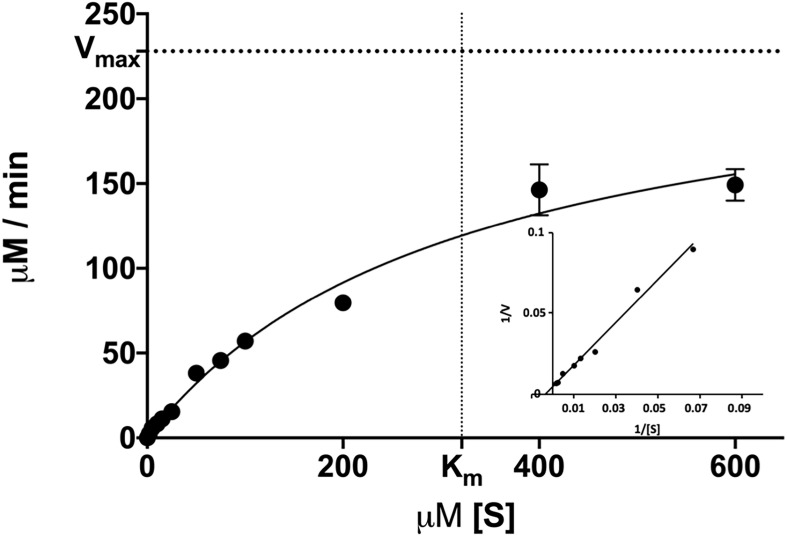
Michaelis-Menten kinetics were determined at various substrate concentrations (2–600 μM) of *p*-nitrophenyl butyrate. The V_max_ of DS-007 was determined to be 238,86 ± 5.30 μM/min and the K_m_ was determined to be 321.69 ± 11.27 μM. The k_cat_ is 398.11 s^–1^ and the k_cat_/K_m_ is 1.24 × 10^6^ s^–1^ mol^–1^. Standard deviation from three independent experiments is shown.

We then determined the enzymatic parameters of DS-007 at its optimal conditions. Michaelis-Menten kinetics were determined at 55°C and pH 9.5 by measuring the hydrolysis activity at various substrate concentrations (2–600 μM) of *p*-nitrophenyl butyrate and an enzyme concentration of 10 nM. V_max_ of DS-007 was determined to be 238.86 ± 5.30 μM/min and K_m_ was determined to be 321.69 ± 11.27 μM. The turnover number k_cat_ of DS-007 was 398.11 s^–1^ and its catalytic efficiency k_cat_/K_m_ is 1.24 × 10^6^ s^–1^ mol^–1^ (Figure 5).

### DS-007 Is Temperature Stable

**FIGURE 6:**
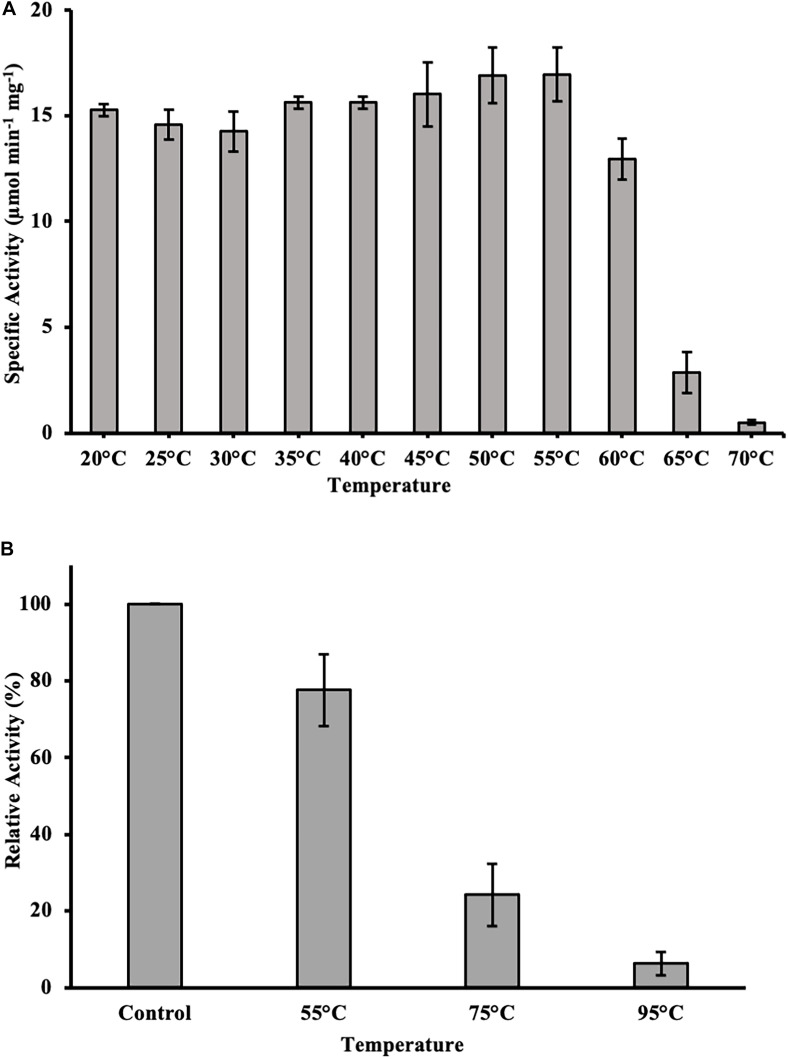
**(A)** Heat stability of DS-007. After incubation of DS-007 for 1 h at 60°C, it still retains around 70% of its initial activity. Incubation at higher temperatures caused a more severe loss of activity. **(B)** Long term heat stability and refolding capacity. Incubation at 55°C for 18 h had a small impact on the activity of DS-007 in an in-gel assay when compared to room temperature (Control). The 75°C sample showed a decrease in activity (25% of the room temperature control). It seems that DS-007 recovers in the refolding process of the activity assay in-gel, thus showing lipolytic activity in the zymogram, even after incubation for 18 h at 75°C. Similarly, DS-007 incubated for 18 h at 95°C still shows detectable, albeit low lipolytic activity in-gel. The average and standard deviation of three independent experiments is shown.

To test for temperature stability, the enzyme was incubated for 1 h at varying temperatures between 20 and 70°C in 50 mM sodium phosphate buffer pH 9.5. Subsequently, an activity assay was done with *p*-nitrophenyl butyrate (C_4_) as substrate at optimal conditions. DS-007 showed high temperature stability, after 1 h incubation at 60°C it retained around 70% activity in comparison to optimal conditions. Temperatures higher than 65°C drastically decreased the activity to less than 5% within 60 min (Figure 6A). We then investigated DS-007’s long time temperature stability and refolding capacity by incubating the enzyme for 18 h at room temperature (control), 55, 75, and 95°C in 50 mM sodium phosphate buffer pH 9.5. Subsequently an in-gel assay with 4-methylumbellyferyl butyrate was performed and screened for fluorescent bands at the position of the enzyme. After incubating DS-007 at 55°C for 18 h, it still showed 70% activity, which decreased to 25% activity when incubated at 75°C (Figure 6B). Even after 18 h at 95°C, the sample still showed residual lipolytic activity.

### DS-007 Shows Tolerance Toward Organic Solvents

**FIGURE 7:**
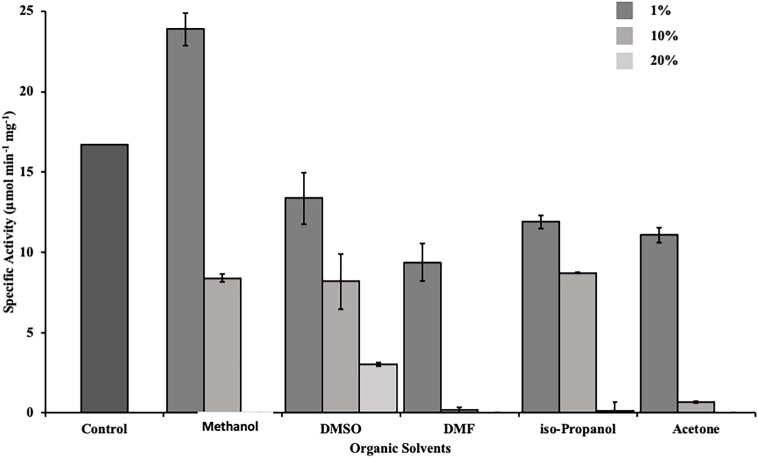
Activity of DS-007 was determined by activity assays in the presence of different organic solvents [1–20% (v/v)] at optimal conditions. DS-007 shows moderate tolerance toward organic solvents. At a concentration of 1% (v/v) of methanol, the activity of DS-007 increased by approximately 40% when compared to control conditions. Even at 10% methanol, DMSO or iso-propanol DS-007 still shows around 50% activity.

Esterases are widely used in the production of fine chemicals and pharmaceuticals. For industrial applications a resilience against organic solvents is desired (Fazary and Ju, 2008). Therefore, we determined the activity of DS-007 in the presence of commonly used organic solvents. DS-007 shows a moderate tolerance toward organic solvents at a concentration of 1% (v/v). In fact, it still shows 65% activity in the presence of 1% acetone. One percent of methanol increases the activity of DS-007 by 40% in comparison to the optimum conditions without solvent. In the presence of 10% methanol, DMSO or isopropanol it still shows around 50% of its initial activity. Ten percent of DMF and acetone diminish DS-007’s activity to less than 10% (Figure 7).

### Several Metal Ions and Detergents Increase Activity of DS-007

**FIGURE 8:**
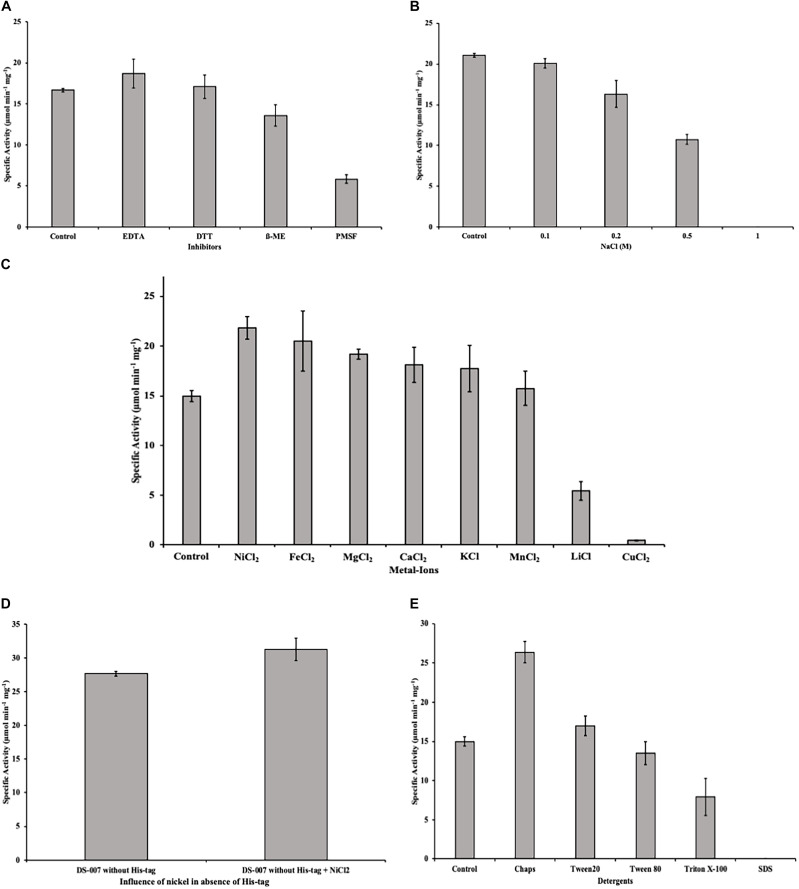
**(A)** Inhibitors like EDTA and DTT at a concentration of 1 mM had no effect on the esterase activity of DS-007. Even PMSF only decreased the activity to 30%. **(B)** Incubating DS-007 with high concentrations of NaCl showed a significant decrease to 50% activity at 0.5 M NaCl. 1 M NaCl led to complete loss of activity. **(C)** Metal ions in a concentration of 1 mM increased the activity of DS-007 by up to 40% (Ni^2+^). Only Li^+^ and Cu^2+^ reduced the activity to 35 and 3%, respectively. **(D)** The effect of Ni2+ is independent of the presence of the hexahistidine tag. Tag-less DS-007 was more active than DS-007 with hexahistidine tag in the absence of Ni^2+^and shows a comparable increase in activity when 1 mM Ni^2+^ was added. **(E)** Detergents like Chaps and Tween 20 [1% (v/v)] increased the activity of DS-007, with Chaps increasing it 1.75-fold. SDS completely inhibited DS-007.

Some unspecific inhibitors like EDTA and DTT at a concentration of 1 mM had no significant effect on the esterase activity of DS-007. However, β-mercaptoethanol decreases the specific activity by 20% and PMSF, a compound that can irreversibly modify serine residues, as the one found in DS-007’s active site, caused the activity to decrease to 30% of its initial value (Figure 8A).

Incubating DS-007 for 1 day with different NaCl concentrations lead to a significant decrease to around 50% activity in the presence of 0.5 M NaCl, while 1 M NaCl led to a complete loss of activity (Figure 8B). Divalent and monovalent metal ions can also affect the esterase activity (Nachmansohn, 1940; Gupta et al., 2004). The addition of 1 mM Ni^2+^, Fe^2+^, Mg^2+^, Ca^2+^, K^+^ and Mn^2+^ all lead to an increase of the hydrolyzing activity with Ni^2+^ having the biggest effect with an increase of up to 40% compared to control. Only Li^+^ and Cu^2+^ reduced DS-007’s activity to 35 and 3%, respectively (Figure 8C). The inhibition of lipases and esterases by copper is a known phenomenon (Hiol et al., 2000). The increasing effect of Ni^2+^ seems not to be an artifact due to the presence of a hexahistidine tag in the recombinantly purified protein. DS-007 without hexahistidine tag but shows an increase in activity of approximately 10% compared to DS-007 without hexahistidine tag and the activity of the tag-less enzyme is further enhanced by the addition of Ni^2+^ (Figure 8D).

However, detergents like CHAPS and Tween 20 [1% (v/v)] increased the activity of DS-007, with CHAPS increasing it 1.75-fold. Tween 80 had no significant effect, whereas Triton X-100 led to a decrease (50%) and SDS reduced the activity to virtually zero (Figure 8E), which has already been observed for other esterases (Sukul et al., 2018).

## Discussion

In this study, a new esterase, DS-007, was identified through a functional metaproteomics approach (Sukul et al., 2017) from the Bakreshwar hot spring in West Bengal, India. The metaproteome from an enrichment culture from this spring was screened for its lipolytic activity on a 2D gel. Mass spectrometry then enabled us to determine the protein sequence of DS-007. DS-007 is an alpha/beta hydrolase from *Ralstonia pickettii*, which has not been biochemically characterized yet. A predicted structure revealed a catalytic triad with Ser-121, Asp-171, and His-198 in close spatial proximity and a characteristic pentapeptide motif GxSxG indicative of enzymes with lipolytic activity (Mala and Takeuchi, 2008) and the absence of a lid suggested to us that DS-007 is an esterase. Our biochemical assays indeed showed that DS-007 has activity toward shorter chain length substrates (≤ C_8_) (Verger, 1997). DS-007 has a thermophilic temperature preference with its optimum at 55°C. The temperature of the hot spring where the samples were taken was 60°C, showing the adaptation to the habitat. However, the free enzyme DS-007 shows only little activity at temperatures higher than 65°C, although it retained significant residual activity in-gel even after 18 h of incubation at 75 or 95°C, pointing toward immobilization as a valid strategy to stabilize DS-007. Other thermostable lipases, e.g., from *Geobacillus* sp. still show detectable activity up to 90°C (Hebin and Xiaobo, 2005).

DS-007 is an alkaliphilic enzyme with an optimum pH at 9.5. A pH of 10.0 on the basic side decreases the activity to 50% as does pH 8.0 on the acidic side. Compared with other known esterases, the pH profile of DS-007 is more on the basic side of the spectrum, but its pH range is fairly typical (Yun-Jung et al., 2006). Both, pH and temperature optima of the enzyme, were found to be similar to those of the original habitat. With a K_m_ of 321.69 ± 11.27 μM, a k_cat_ of 398.11 s^–1^ and a catalytic efficiency k_cat_/K_m_ of 1.24 × 10^6^ mol^–1^ s^–1^ it has parameters typical for esterases and the catalytic efficiency is comparable with other thermophilic esterases (Manco et al., 1994; Kakugawa et al., 2006; Yu et al., 2010). As known from literature many esterases are activated or inhibited by metal ions. For example, acetylcholinesterase is activated by Mg^2+^, Ca^2+^, Mn^2+^ and Na^+^ and inactivated by Ni^2+^ and Cu^2+^ (Tomlinson et al., 1981). Metal ions also had a significant effect on DS-007, increasing its activity by up to 40% with Ni^2+^ or Fe^2+^ cations. For some esterases, iron can increase the activity (Szczyrba et al., 2014). Only two cations decreased the activity of DS-007. Morimoto et al. (2006) described that lithium can have inhibitory effects on esterases and lipases, presumably by binding close to the catalytic site and thereby disturbing the structure, explaining why Li^+^ reduces the enzyme’s activity to 35% (Gangadhara et al., 2010). Cu^2+^ cations reduced the activity to 3%, which is an already known phenomenon (Hiol et al., 2000). With the exception of PMSF, all inhibitors showed negligible effects. As mentioned before, DS-007 is an esterase containing a catalytic triad with Ser-Asp-His in the active side, whereby the serine is strongly conserved in a Gly–X–Ser–X–Gly motif (Derewenda and Derewenda, 1991; Bornscheuer, 2002b). PMSF is a serine hydrolase inhibitor, whose sulfonyl group undergoes a nucleophilic attacked by the hydroxyl group of the serine. This leads to an irreversible sulfonylation of the serine in the catalytic triad. But DS-007 was just inhibited up to 70%. One explanation could be that sterical reasons prevent binding of PMSF to the serine in the active site. However, detergents like CHAPS and Tween 20 [1% (v/v)] increased the activity of DS-007, with Chaps increasing it 1.75-fold, whereas Triton X-100 leads to a decrease and SDS reduced the activity to 0%, which was already observed for other esterases (Sukul et al., 2018). The fact that DS-007 showed activity on an SDS-PAGE after washing in 50 mM sodium phosphate buffer demonstrates that the protein can be effectively refolded. The immobilization of DS-007 on a polyacrylamide resin thus seems to be feasible, potentially providing further enzyme stability. Because many esterase-catalyzed reactions in biotechnological industry are performed in non-aqueous solutions we tested the effect of different organic solvents on the activity of DS-007. All tested organic solvents showed inhibitory effects on DS-007, except methanol. One percent of methanol increased the activity of DS-007 by 40% in comparison to optimal aqueous conditions. Even with 10% methanol DS-007 still shows 50% activity. A 10% concentration of DMSO and isopropanol reduces the activity to 50% at most. Only 10% of DMF and acetone diminish the activity to less than 10%. For an esterase, DS-007 shows moderate tolerance against organic solvents.

Our results further demonstrate the usefulness of the functional metaproteomic approach (Sukul et al., 2017). Choosing an appropriate microbial habitat for screening, we were able to directly discover a thermo-stable and solvent-tolerant enzyme from the metaproteome without the need for protein engineering. With appropriate in-gel substrates, this method has also the potential to identify enzymes from a variety of other classes. The enzyme we discovered with this approach, DS-007, was successfully expressed in *E. coli*, had optimal activity at 55°C and pH 9.5 with a preference for short chain length substrates (≤ C_8_), typical for esterases. DS-007 also shows appropriate resilience against organic solvents.

## Data Availability Statement

The original contributions presented in the study are included in the article/supplementary material, further inquiries can be directed to the corresponding author/s.

## Author Contributions

DS and LL designed the experiments and wrote the manuscript. PS, TM, and SM collected the hot spring samples in India. DS and PS performed the 2D-gel electrophoresis. DS purified DS-007. DS and YY performed the enzymatic characterization of DS-007. SS and JB performed mass spectrometric experiments and provided assistance during 2D gel electrophoresis. All authors contributed to the article and approved the submitted version.

## Conflict of Interest

The authors declare that the research was conducted in the absence of any commercial or financial relationships that could be construed as a potential conflict of interest.

## Abbreviations

DMF: dimethylformamid
DMSO: dimethyl sulfoxide
DTT: dithiothreitol
EDTA: ethylenediaminetetraacetic acid
PMSF: phenylmethylsulfonyl fluoride
*p*MUB: *para*-methylumbelliferone butyrate
*p*NP: *para*-nitrophenol.
